# Sentiment analysis of hotel online reviews using the BERT model and ERNIE model—Data from China

**DOI:** 10.1371/journal.pone.0275382

**Published:** 2023-03-10

**Authors:** Yu Wen, Yezhang Liang, Xinhua Zhu

**Affiliations:** 1 Guilin Tourism University, Guilin, China; 2 Institute of Culture and Tourism, Guilin Tourism University, Guilin, China; 3 School of Computer Science, Guangxi Normal University, Guilin, China; Jeonbuk National University, REPUBLIC OF KOREA

## Abstract

The emotion analysis of hotel online reviews is discussed by using the neural network model BERT, which proves that this method can not only help hotel network platforms fully understand customer needs but also help customers find suitable hotels according to their needs and affordability and help hotel recommendations be more intelligent. Therefore, using the pretraining BERT model, a number of emotion analytical experiments were carried out through fine-tuning, and a model with high classification accuracy was obtained by frequently adjusting the parameters during the experiment. The BERT layer was taken as a word vector layer, and the input text sequence was used as the input to the BERT layer for vector transformation. The output vectors of BERT passed through the corresponding neural network and were then classified by the softmax activation function. ERNIE is an enhancement of the BERT layer. Both models can lead to good classification results, but the latter performs better. ERNIE exhibits stronger classification and stability than BERT, which provides a promising research direction for the field of tourism and hotels.

## 1. Introduction

With the development of information technology and the increasing improvement of online booking and payment functions, online review data has become one of the important sources of competitive intelligence for enterprises [1]. In the hotel industry, more and more customers are booking hotel rooms through hotel websites or related online platforms, such as Hotels.com, Booking.com, and Ctrip [2]; more and more hotel customers are seeking advice from each other through online online reviews [3], and the effectiveness of traditional hotel advertising is declining, while the online The impact of hotel reviews is on the rise [4]. Online reviews are posted by customers who have experienced the hotel’s services, so for potential customers, online hotel reviews are more persuasive than the hotel’s own advertisements [5]. Online reviews will significantly influence customer attitudes, purchase decisions, and thus company performance [6–9]. A report on the Siteminder hotel services platform 2021 shows that more than two-thirds of of global travelers use travel review sites before booking, with 93% saying online reviews influence their booking decisions and 79% reading 6 to 12 reviews before making a purchase decision [10].

Therefore, online reviews have important commercial value for improving hotel service quality and attracting consumer traffic, and it is important to improve the classifiability and accuracy of sentiment analysis of hotel online reviews. In practice, through the sentiment analysis of hotel’s online reviews, on the one hand, hotel managers can obtain customers’ experience evaluation and collect positive and negative comments to improve the personalized service and quality of the hotel [11]. In addition, by understanding customers’ emotional expressions and emotional analysis, they can capture the real inner emotions of users and understand the inner needs of customers, thus enabling hotels to better and more accurately capture their target groups, meet different needs and improve the core competitiveness of hotel brands. On the other hand, in the context of big data, sentiment analysis as an important tool can also help potential customers to make effective consumption decisions [12].

Emotion analysis based on hotel online reviews is a relatively new research topic that provides an intelligent application direction for personalizing customers’ accommodation needs. In addition, it creates space for developing high-quality tourism and improving tourist accommodation satisfaction [13]. However, there are many aspects of hotel online comment emotion [14], such as hotel price, star rating, location, room facilities, meals, staff and age group of consumers. For example, because language has multiple meanings, with positive ratings, negative ratings, and neutral ratings, it adds to the difficulty of collecting valid hotel online comment emotion [15]. However, it is impossible to accomplish this simply by manually organizing the huge amount of online reviews, and deep mining with the help of computers is needed to make more scientific decisions. To meet this challenge, some scholars have combined machine learning methods with sentiment analysis theories to classify the sentiment of online reviews in hotels by means of classification models, namely, plain Bayesian, K-nearest neighbor, support vector machine, logistic regression, and random forest [16], in order to achieve more accurate sentiment analysis. With the development and application of machine learning theory, the advantages of neural network technology in deep learning are becoming more and more obvious, and the fusion of multiple models has become a favorable guarantee for the accuracy of text sentiment classification, which can well solve the phenomenon of multiple meanings of the word and capture the depth characteristics of words in hotel web reviews [17].

This paper presents the results of crawling 16,000 customer reviews of Ctrip hotels in China using the BERT model to classify emotions in comment data. This approach can tap into the true feelings and internal needs of customers so that hotels can better and more accurately lock target groups, meet differentiated customer needs through market segmentation, and implement differentiated brand development strategies, thereby enhancing the core competitiveness of hotel brands. In summary, this paper makes four main contributions as follows:

1. Among the large number of review texts written by customers, extreme positive and negative emotions are few and easy to distinguish, while review texts with neutral emotional tendencies are abundant and have ambiguous sentiment tendencies, resulting in low customer ratings. Therefore, instead of using customer ratings for sentiment classification, this paper uses a classification model to classify customer review texts into two sentiment categories: those with rating values greater than or equal to 3 are classified as positive, and those with ratings less than 3 are classified as negative. All review texts are classified into positive and negative sentiments only, and no neutral sentiments are assigned. This improves the accuracy of text classification to a certain extent, and the hotel will only process positive and negative sentiments and will not waste time on neutral comments.
2. The classifier based on BERT model fusion and BERT-enhanced ERNIE are used to analyze the emotion of hotel customer review text. Both models can better learn the semantic information between contexts and resolve polysemy in the text. The trained language model is used to transform words directly into vectors with word granularity.
3. Improved input processing is applied to the BERT model. At the fine-tuning stage, the BERT model is fused with a CNN and RNN to train the hotel review text dataset. Compared with the traditional model, BERT achieves a better classification effect, which verifies the effectiveness of the BERT model for hotel review text classification.
4. The BERT model is deeply analyzed, and corresponding improvements are made to address the shortcomings of the BERT model by using the enhanced ERNIE model. The ERNIE model has better performance and stability than the original BERT model.

## 2 Related research

Emotion analysis evaluates text based on emotion color. Implied emotion offers an important supplement to the understanding of the content and viewpoint of text, which is the core research field of NLP. Due to the development of the internet, people like to comment and interact with others on social platforms, resulting in a large number of short comments. Although short, these online comments have a rich emotional vocabulary. However, due to the irregularity of online short comment sentences and the multilayered meaning of words, it is difficult for researchers to analyze the emotions of short comments. Google AI Language Department proposed a new language representation model of BERT from Transformers’ bidirectional encoder representation [18]. The BERT model proves the importance of bidirectional pretraining for language representation, which is a typical mask language model (MLM); it also has strong universality; therefore, it can be fine-tuned to suit most NLP tasks, such as sequence annotation and text classification [19]. In recent years, the BERT model has become the most advanced and popular machine learning model in academia and industry. It can be used to accomplish multiple NLP tasks without manual supervision, such as supervised text categorization. At the same time, it can flexibly handle any type of corpus with excellent results. Gonz â alez-Carvajal et al. (2020) experimentally proved the superiority of BERT and its independence from the characteristics of NLP problems [20]. By combining different parallel blocks of a single-layer deep convolutional neural network (CNN) with different kernel sizes and filters with BERT, a BERT-based deep learning method (FakeBERT) was proposed for the detection of fake news in social media [21]. Shobana et al. (2022) improved self-attention mechanism is added with BiLSTM for focusing on significant words in the context. To enhance the performance of Bidirectional Long Short-Term Memory, the weight parameters of Bi-directional LSTM are optimally selected by using APSO algorithm [22].

Although the BERT model is widely used to study social media emotion analysis, few scholars have applied this model to the emotion analysis of tourism industries, such as hotels and restaurants, which is a vital and cutting-edge field. At present, an increasing number of travelers prefer to share their experiences and feelings of hotel accommodation through social media and travel network platforms, and many potential consumers make decisions after checking these comments. The rapid growth of large sets of unstructured text data and the proliferation of tools for analyzing them have brought great opportunities and challenges to text mining research. Some scholars have performed comparative analyses by using naive Bayes polynomials, order minimum optimization, supplementary naive Bayes and composite hypercubes. To find a machine learning algorithm suitable for framework classification components, an emotion analysis framework with opinion mining is designed, which automatically prepares emotion datasets for training and testing to extract fair opinions of hotel services from comments [23]. Some scholars have performed data collection, data preprocessing, feature engineering composed of word frequency-inverse document frequency, feature generation and feature selection based on Doc2Vec, random forest classification, data analysis and data visualization to obtain more decision-making information for the analysis and exploration of the attitudes and emotions expressed in reviews, thereby improving the service quality of luxury hotels with marketing insights [24]. Existing fine-grained emotion analytical methods cannot be used to perform implicit aspect-level term extraction. To understand the preferences of hotel customers, an unsupervised aspect-level emotion analytical method is proposed by integrating word embedding, co-occurrence and dependency resolution, in which the implicit hotel attributes are considered. In other words, a method based on the overall emotional value of hotel attributes is used to understand customer preferences to support hotel service analysis [2]. Wang et al. (2021) retrieved more than 120000 online reviews related to hotels and proposed a new seven dimensional evaluation system based on datasets, applying Bert model [25]. Zhuang et al. (2021) studied how to build a multi criteria recommendation system using a fine tuned Bert model to recommend suitable target customers for hotels [26].

In fact, finding a suitable hotel that matches users’ needs and affordability is a complex decision-making process. The integration of hotel online review big data and the BERT model’s novel technology has created a new research direction that provides a higher accuracy of emotion analysis. Due to the short text data of comments, the traditional emotion classification algorithm fails to understand the deep meaning of words and solves the problem of polysemy. In contrast, BERT can better capture deep features and identify words accurately, better express the deep meaning of words and achieve better classification results. For example, Lu et al. (2020) have proposed Bi-GRU Sentiment Classification for Chinese Based on Grammar Rules and BERT [27]. Tian et al. (2022) have used the SemEval 14 Restaurant Review dataset, combined with the capsule network and BRET, and proposed the baseline model capsnet Bert to prove the polarity of accurately extracting emotional features [28]. Ray et al. (2021) have used a set of BERT and random forest models to establish a set of different transfer-learning models for different text features of reviews, thereby classifying the emotions of hotel reviews and designing a hotel recommendation system based on online reviews in English [29].

Sun et al. (2019) have proposed a model called ERNIE (enhanced representation through knowledge integration) by using knowledge masking strategies.They confirmed that the model has better universality and adaptability in recognizing longer semantics [30]. Zhang et al. (2019) have used text corpus and knowledge map to train Ernie model. Experiments show that the model can make full use of vocabulary, syntax and knowledge information at the same time, and has made significant progress in various knowledge driven tasks [31]. To better classify hotel online reviews, this paper uses the classifier based on a BERT model fusion and a BERT-enhanced ERNIE to analyze the emotion of hotel customer review texts, fine-tunes the BERT pretraining model, and innovatively proposes the ERNIE model to classify hotel review texts. The ERNIE model is experimentally shown to have the highest accuracy with a better classification effect.

## 3. Research design of emotion classification using the BERT model

### 3.1 BERT word vector model

**Fig 1:**
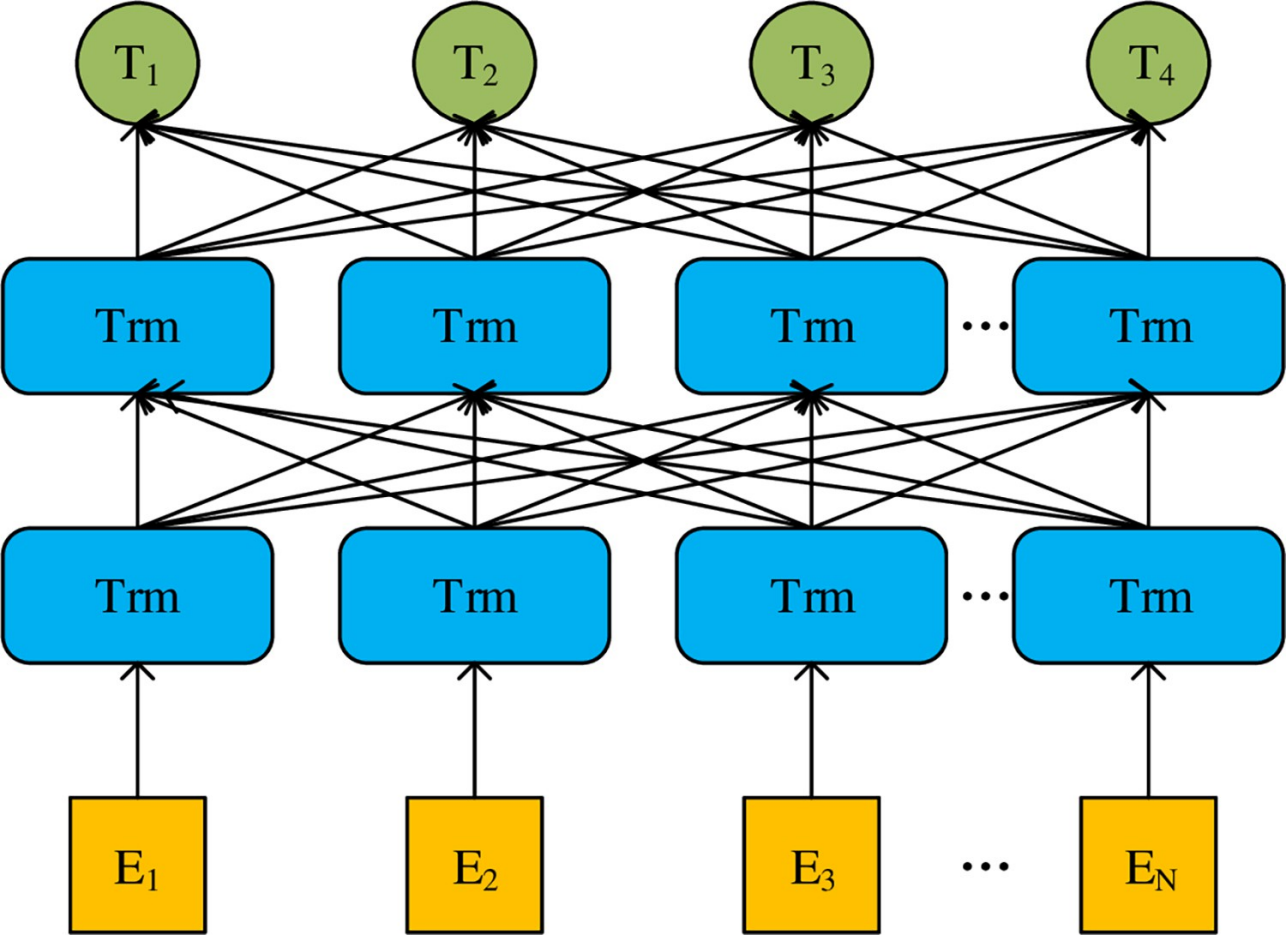
BERT model structure diagram.

The BERT (bidirectional encoder representation from transformers) model is a pretraining language model based on deep learning, which was first proposed by Google AI. As a neural network language model, a pretraining language model can directly train many untagged texts, which is applicable for various downstream tasks of natural language processing, such as text classification, text annotation and automatic question answering. The advantage of the pretraining language model is that it only needs to be fine-tuned when facing different downstream tasks, without retraining the model. In addition, as a bidirectional language model, the main structure of the BERT model is the encoder part of the transformer model. The two-way language model is embodied in the pretraining task of masked LM, in which words are input into the model in parallel and the masked part is predicted by using context semantic information. Google AI has trained two BERT models for users to use directly, divided into a BERT-based model and a BERT-large model according to parameter size. The BERT model structure diagram is as follows (see Fig 1):

### 3.2 BERT input processing

**Fig 2:**
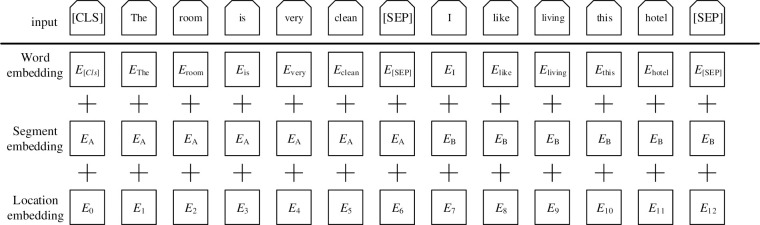
BERT input sequence structure diagram in the model.

The text sequence E input into the BERT model will be decomposed into three parts, i.e., token embeddings, segment embeddings and position embeddings. The text sequence T understandable by the model will be obtained by the addition of vectors with the same dimensions in the three parts. This process can be visualized as shown in Fig 2.

### 3.3 ERNIE model

**Fig 3:**
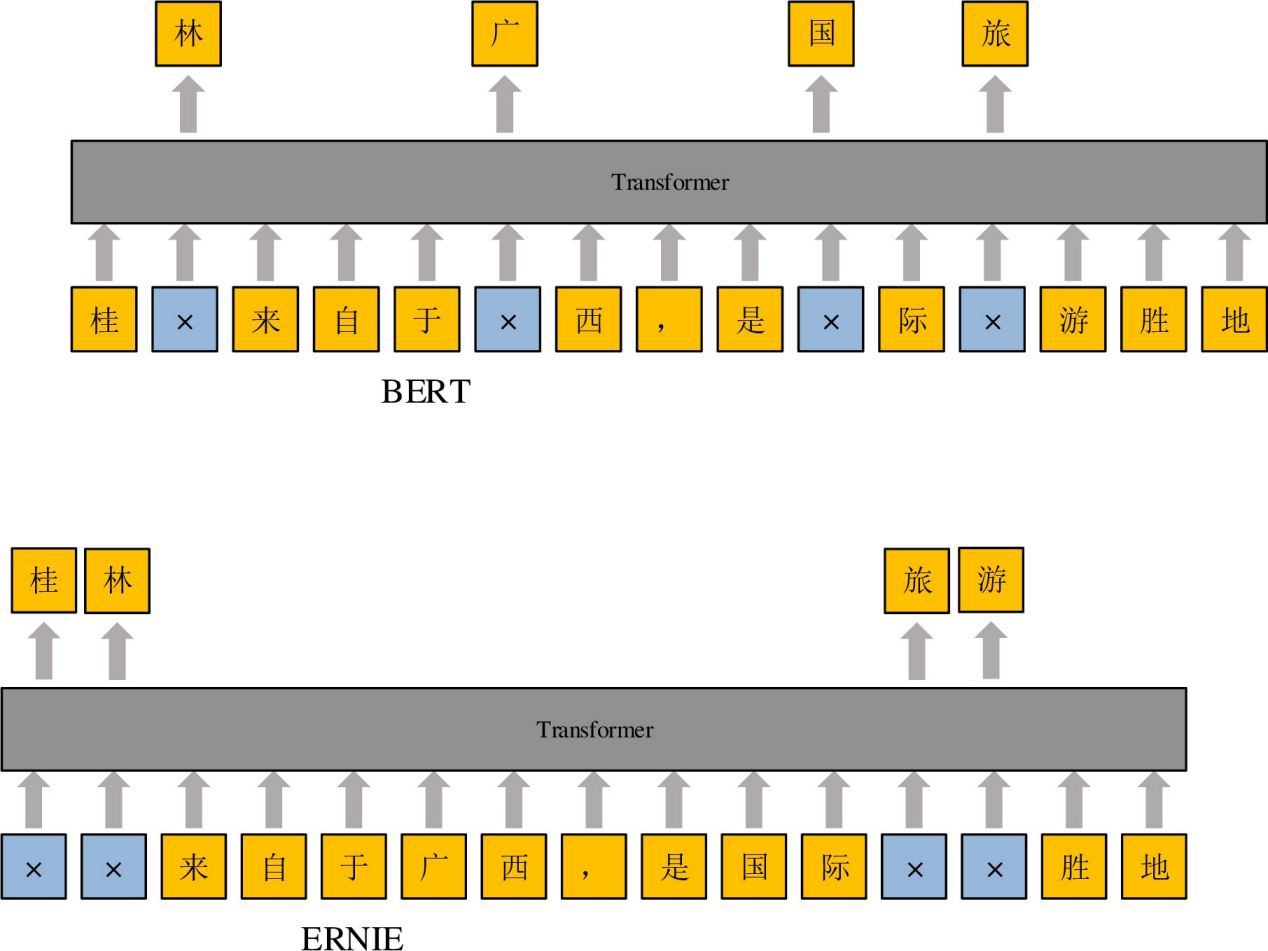
Differences in the mask strategies between the BERT model and ERNIE model.

The ERNIE model is based on the BERT model structure, which makes full use of lexical, syntactic and semantic information. Compared with the BERT model, the improvement of the ERNIE model lies mainly in the mask mechanism. Differences in mask strategies between the BERT model and ERNIE model are shown in the figure. The modeling object of the BERT model focuses mainly on the original language signal, while the lexical structure, syntactic structure and semantic information of the training data are not fully used to learn modeling. When training Chinese texts, the BERT model randomly covers up to 15% of words for prediction. Based on this forced cover-up, the relationship between the words “Gui” and “Lin” is separated and that between the words “Guilin” and “Tourism” is blurred. The mask language model adopted by the ERNIE model is a mask mechanism with prior knowledge. By modeling semantic information, such as words and phrases, the relationship between “Guilin” and “tourism” is obtained. In addition, it is learned that “Guilin” is the “location” of “Guangxi”, and “Guilin” is called a “tourist resort”, inferring that the concealed word at this time is “Guilin”, which makes the model learn the semantic representation of the complete concept, as shown in Fig 3:

### 3.4 Text preprocessing

The original corpus often contains a large amount of redundant information, which is not suitable for training directly and usually needs to be cleaned first. In the synchronous binary classification model proposed in this paper, the question text in the hotel comment corpus contains many irregular contents, such as special symbols, garbled codes, and stop words. It is then necessary to preprocess the comment sentences to mitigate the influence of noise and redundant information to ensure the text in the emotional corpus achieves the best effect. The length of hotel comment text is different; therefore, it needs a fixed length or dimension input. To satisfy this requirement, this paper sets the size text of pad_size to fill zero and truncate. After data preprocessing, the experimental data of this paper are obtained. Part of the text corpus is shown in Table 1 below.

**Table 1:** Word breakdown of hotel reviews.

Serial number	Comment content	Affective classification
1	The room facilities are very good, the service is good, and the transportation is convenient	1
2	Disappointed, small broken sink, worn and dirty everywhere	0
3	The room is messy, the air conditioner leaks and makes noises	0
4	The room environment is good, the breakfast is delicious, and the cost performance is high	1
5	Good service, elegant environment, quick order processing	1

### 3.5 Experimental design

**Fig 4:**
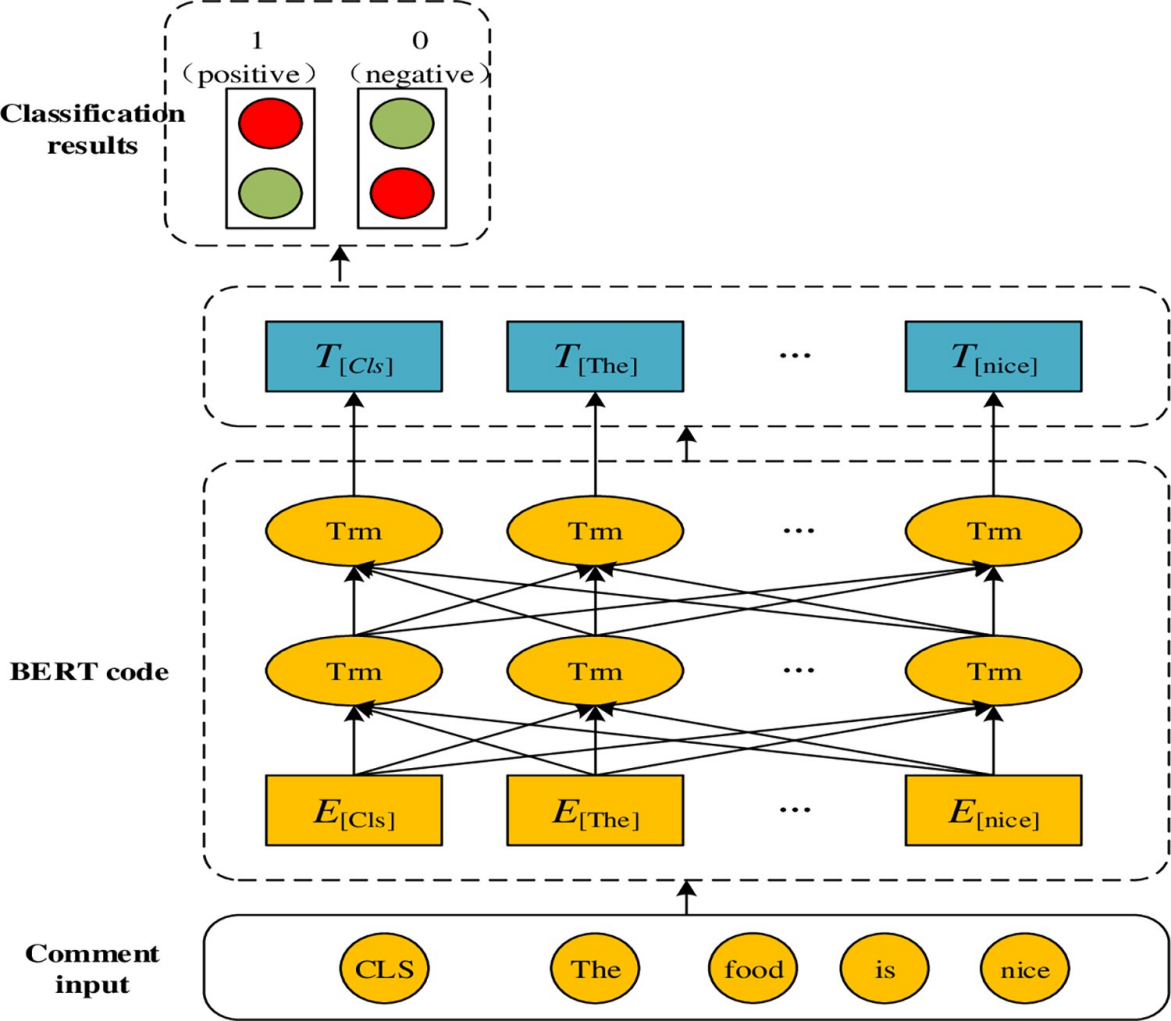
BERT classification diagram.

Treat an input comment text as a single sentence, and add the special mark of [CLS] to the first bit to obtain the input token sequence E = {E1, E2,…, En}. First, use BERT as the embedding layer to encode the input token and obtain the vector representation T = {T1, T2,…, Tn} of the output token, where T1 is still the [CLS] tag, corresponding to the hidden layer state of the output, which is not the feature of the entire single sentence only. Then, the classifier layer is used to output the classification result, as shown in Fig 4:

## 4 Experimental analysis and results

### 4.1 Experimental dataset

The experimental data source of this paper is the dataset of reviews of major hotels in China. The data set consists of 16000 hotel reviews, including 8000 positive reviews and 8000 negative reviews, with a positive to negative sample ratio of 1:1. When dividing the data set, the training set takes 10000 reviews, including 5000 positive reviews and 5000 negative reviews; the test set takes 3000 reviews, including 1500 positive reviews and 1500 negative reviews; the verification set takes 3000 reviews, including 1500 positive reviews and 1500 negative reviews. The ratio of training set, test set and check set is 3.3:1:1. Therefore, the data set used for this paper is a balanced data set.

### 4.2 Experimental design and parameter selection

**Fig 5:**
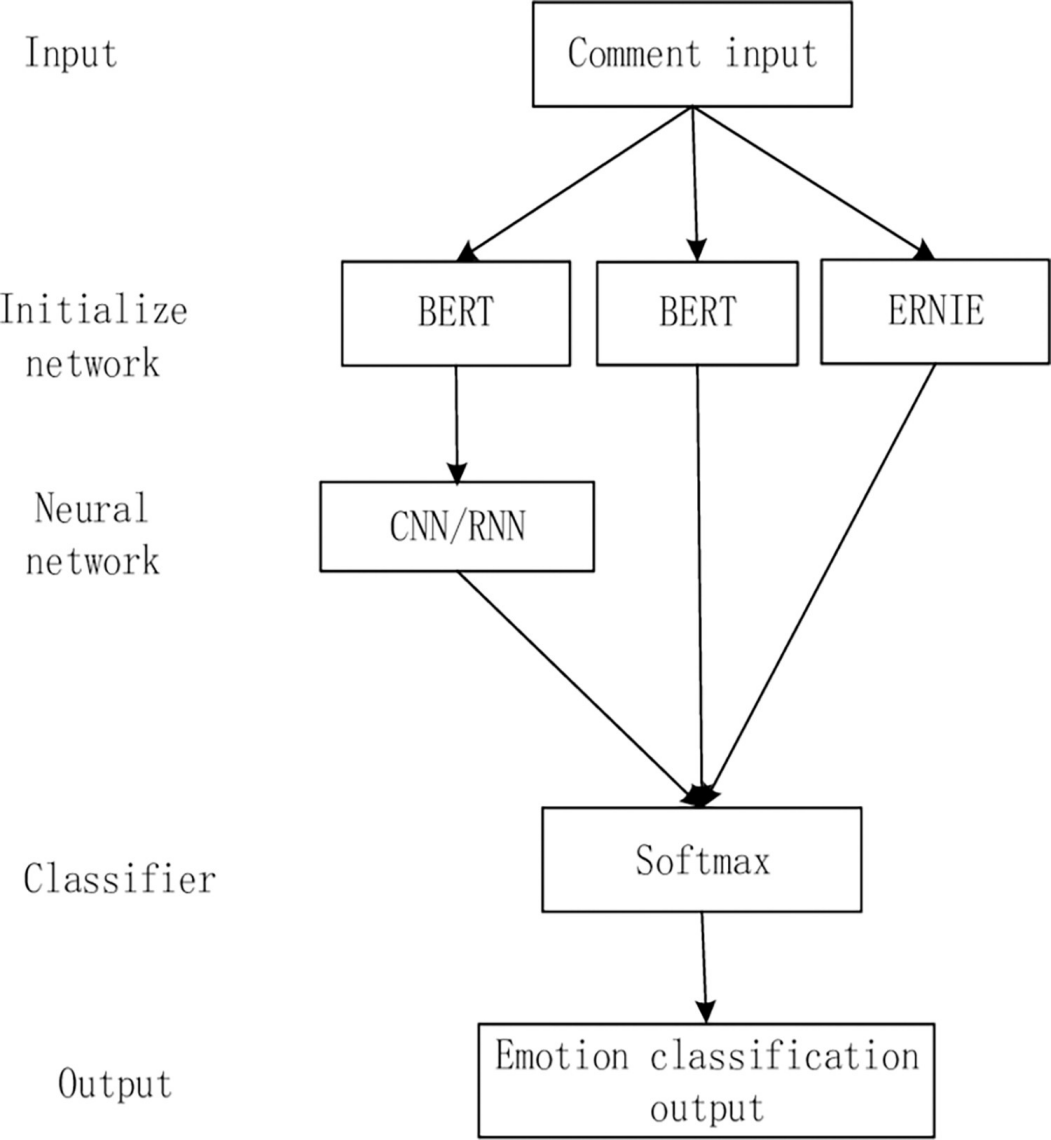
Design of the classification model.

In this paper, the BERT model suitable for simplified and traditional Chinese is used to transform hotel customer comment text after data processing. Different from other Chinese word vector models, the BERT model can segment the text with word granularity. A variety of open-source BERT models suitable for Chinese text can be downloaded from the GitHub website. In this paper, the Chinese_roberta_wwm_large model is selected. Whole Word Masking (wwm) is the whole word covering, that is, covering all Chinese characters that constitute the same word, which contributes to accurate classification. The BERT model is a simple pretraining language model with its input and output in vector form. To accomplish the task of text classification, this paper adds a softmax layer to the output layer for simple emotion classification of input vectors. The specific design is shown in Fig 5 below:

In this experiment, the model with high classification accuracy is obtained by adjusting the values of the above three parameters without considering the running time and memory. In the BERT pretraining model, the default preset values of the three parameters are given as follows: the value of epochs is set to 5; the value of batch_size is set to 128; the value of pad_size is set to 32; the value of learning_rate is set to 1e-5; and the value of hidden_size is set to 768, as shown in Table 2 below:

**Table 2:** Parameter configuration table.

Parameter	Parameter name	Parameter value
***Epochs***	Training time	5
***Batch_size***	Batch size	128
***Pad_size***	Sentence length	32
***Learning_rate***	Learning rate	1e-5
***Hidden_size***	Number of hidden layers	768

### 4.3 Evaluation index

For a basic binary classification problem, the results can be classified into TP, FP, FN and TN. TP represents a positive sample and the prediction result is correct, FP represents a negative sample and the prediction result is incorrect, FN represents a positive sample and the prediction result is incorrect, TN represents a negative sample and the prediction result is correct. The calculation formula of the corresponding accuracy rate, recall rate and F1 value is as follows:

$$\mathrm{Accuracy}=\frac{TP+TN}{TP+FN+TN+FP}$$

$$\mathrm{Recall}=\frac{TP}{TP+FN}$$

$$F_{1}=\frac{2*\;\mathrm{Precision}\;*\;\mathrm{Recall}}{\mathrm{Precision}+\mathrm{Recall}}=\frac{2*TP}{2*TP+FP+FN}$$

In this paper, for the tourism review texts in the experimental corpus, it is hoped that the emotion types predicted by using the model are maximally consistent with the real emotion types. The cross entropy between the real emotion type and the predicted emotion type is used as the loss function in model training. When the loss function is determined, the BP algorithm is used for model training. The specific formula of the loss function in this paper is as follows:

$$Loss(p,q)=-\sum_{i=1}^{n}p(x_{i})\log(q(x_{i}))$$

where *p*(*x_i_*), *q*(*x_i_*) and N represent the number of emotion classifications, the probability value of real emotion types and the probability value of predicted emotion types, respectively. Cross entropy is utilized to measure the approximation degree of the two distributions. The higher the approximation value of the two distributions is, the smaller the cross entropy is and the closer it is to the true emotional type.

### 4.4 Analysis of results

1. Comparison of experimental models

**Fig 6:**
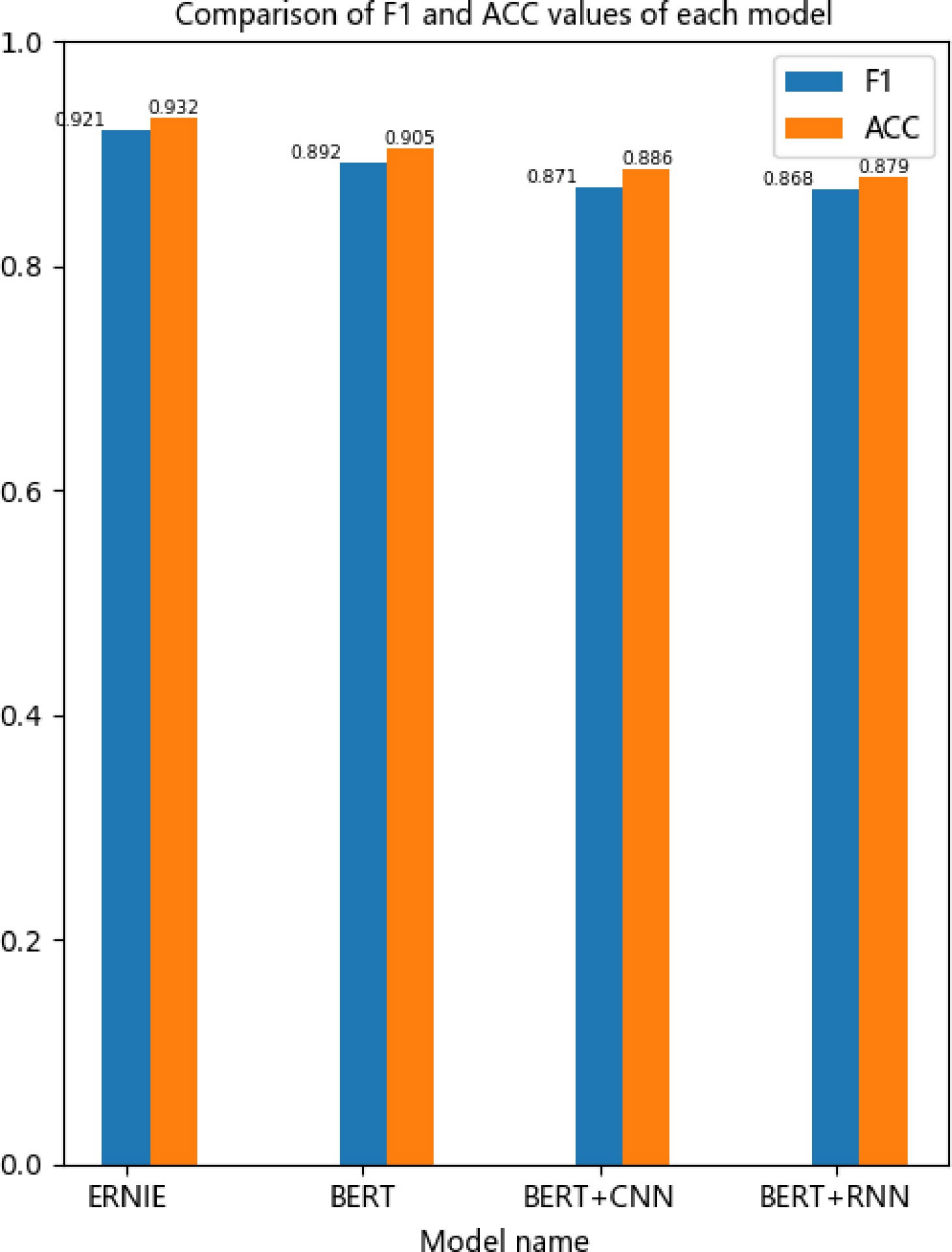
Comparison of F1 and ACC values for each model.

**Fig 7:**
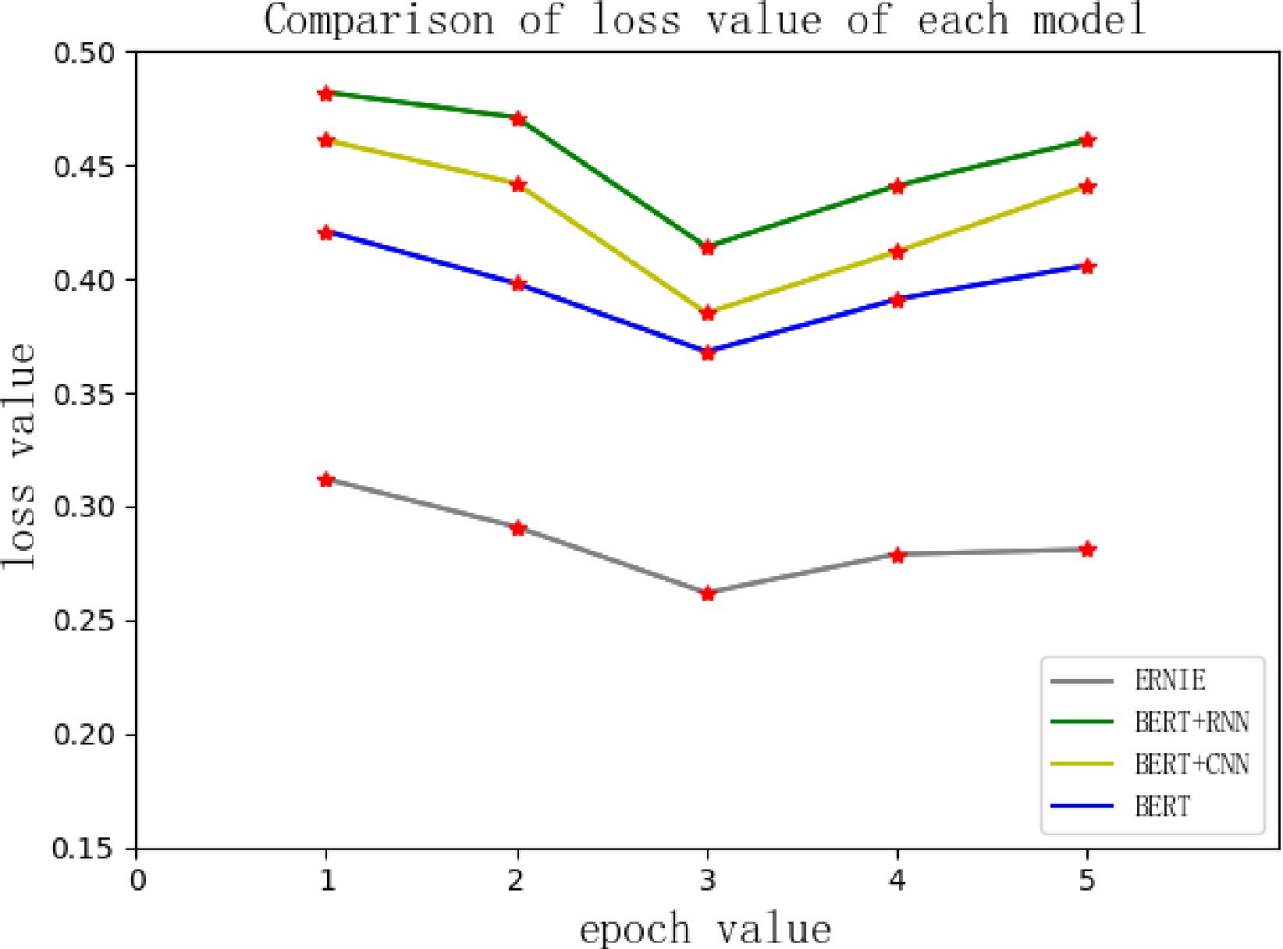
Comparison of the loss value of each model.

In this paper, the hotel review dataset is tested, and the BERT model, BERT fusion CNN, RNN neural network model and BERT-enhanced ERNIE model are compared, as shown in the following figure. Compared with the BERT + CNN and BERT + RNN models, the BERT and ERNIE models performed better in terms of F1 and accuracy ACC. In terms of handling short text classification, the BERT model is sufficiently complex to produce a good classification effect without the addition of a complex neural network. However, the ERNIE model is based on BERT enhancement, which overcomes the issue that BERT cannot learn complete semantics effectively, thus producing a better classification effect, as shown in Fig 6:

The iteration times of this experimental training are set to 5, and useful information can be obtained by observing the change curve of the loss value. When the training of each model starts, the loss value continues to decline, and the accuracy of the model on the verification set continues to improve. However, from the third epoch, the loss value begins to rise, and then the model may have started to overfit, as shown in Fig 7:

2. Analysis of experimental results

The experimental results show that both BERT fusion neural network models and the BERT-enhanced ERNIE model achieved good results in the training process, followed by ERNIE, BERT, BERT + CNN and BERT + RNN. The ERNIE classification model has the highest accuracy, which is mainly because short-text classification tasks tend to use shallow features of language, while BERT is better than ERNIE at capturing deep features, and its potential in this respect is not fully harnessed. The enhanced ERNIE classification model fully improves the vector representation of BERT at the sentence level and thus can effectively learn the complete semantics. The vectors are trained and classified through softmax output, which leads to better results.

## 5. Conclusion

With the rapid development of the mobile internet, Chinese social e-commerce has become a new business model for enterprises to increase their market share. For Chinese hotel services, an increasing number of users have become accustomed to using application software to book room services before and after travel, and the online review data of Chinese hotels are more convincing. The evaluation function of these application software also becomes the link between the hotel and the customer. Specifically, customers express their feelings about their hotel stay to the hotel in the form of comments, and the hotel responds to customers’ comments of satisfaction or dissatisfaction, which provides feedback to the user, thus enabling timely and benign communication between the hotel and the customer. Emotion analysis of customer comments can not only help service software fully understand customer needs but also help potential customers quickly find their desired service products.

In this paper, the hotel review text from China’s ctrip is selected to obtain the experimental dataset through data cleaning, stopping words, text segmentation and emotional part-of-speech tags. In addition, the emotion analytical experiment is carried out by establishing the BERT model. By adjusting the parameters that affect the classification results in the model, the emotion classification model with the best classification effect is established. Comparing the BERT + CNN, BERT + RNN, BERT and ERNIE models, the experimental results show that the ERNIE model has superior accuracy, recall and F1 value for the emotion classification of hotel review texts.

## Supporting information

S1 Data (RAR)
